# Molecular correlates of neural health associate with cardiovascular states

**DOI:** 10.1002/alz.094632

**Published:** 2025-01-09

**Authors:** Anna M. VandeBunte, Bailey L Ortiz, Emily W. Paolillo, Shubir Dutt, Isabel Sible, Brandon Chan, Argentina Lario Lago, Julio C. Rojas, Rowan Saloner, Claire J. Cadwallader, Coty Chen, Valentina E. Diaz, Lana S. Callies, Savannah R. Hallgarth, Miwa Tucker, Joel H. Kramer, Kaitlin B. Casaletto

**Affiliations:** ^1^ Memory and Aging Center, UCSF Weill Institute for Neurosciences, University of California, San Francisco, San Francisco, CA USA; ^2^ University of California San Francisco, San Francisco, CA USA; ^3^ University of Southern California, Los Angeles, CA USA; ^4^ Memory and Aging Center, UCSF Weill Institute for Neurosciences, University of California San Francisco, San Francisco, CA USA

## Abstract

**Background:**

Suboptimal cardiovascular health poses a significant risk for dementia, yet biological links between cardiovascular health and brain function are poorly understood. We examined how plasma markers of astrocytic activation (GFAP), neuronal axon breakdown (NfL), and Alzheimer’s disease (AD) pathology (pTau181) relate to several behavioral and risk indicators of systemic cardiovascular health in older adults along the AD continuum.

**Method:**

209 older adults (59% female; 88% clinically normal; 15% amyloid PET+) completed 30‐day FitbitTM Flex2 monitoring (average daily steps), blood pressure (BP) and heart rate quantification, and plasma assayed for GFAP, NfL, and pTau181 (Quanterix Simoa). Regression models evaluated associations among indicators of cardiovascular health (pulse pressure [systolic BP‐diastolic BP], systolic BP, physical activity, resting heart rate) and plasma markers. Post‐hoc mediation models (1000 bootstrap resampling) evaluated mediational relationships among vascular health indicators and NfL. Models adjusted for age, sex, and BMI.

**Result:**

Elevated pulse pressure associated with higher plasma pTau181 (β = 0.21, p<0.01), NfL (β = 0.19, p<0.01), and GFAP concentrations (β = 0.16, p = 0.01). Higher systolic BP associated with higher plasma pTau181 (β = 0.18, p = 0.03) and NfL concentrations (β = 0.19, p<0.01). Greater average daily steps associated with lower plasma NfL (β = ‐0.15, p = 0.04, but not pTau181 (β = ‐0.10, p = 0.27) or GFAP (β = ‐0.13, p = 0.057). Resting heart rate did not associate with plasma biomarker concentrations (ps>0.50). Given physical activity is a cardiovascular behavior that may support vessel health, we tested a post‐hoc mediation model examining if systolic BP or pulse pressure mediated the association between step count and NfL. Systolic BP, but not pulse pressure, fully mediated the relationship between daily steps and plasma NfL (Indirect Pathway: β = ‐0.04, p = 0.03; Direct Pathway: β = ‐0.11, p = 0.14).

**Conclusion:**

Elevated pulse pressure and systolic BP associate with higher levels of AD pathology, astrocytic activation, and axonal degeneration (pTau181, GFAP, NfL), while greater average daily steps associated with lower levels of neurodegeneration (NfL). Systolic BP may drive the association between physical activity and axonal degeneration, highlighting the importance of BP management in mitigating the effects of neurodegeneration. Studying links among cardiovascular health indicators and molecular brain outcomes could inform precise intervention targets for optimal brain aging, such as promoting the importance of blood pressure control and physical activity engagement.

